# CD4^+^ T helper 2 cell–macrophage crosstalk induces IL-24–mediated breast cancer suppression

**DOI:** 10.1172/jci.insight.180962

**Published:** 2025-01-09

**Authors:** Bo Wang, Yun Xia, Can Zhou, Yuhan Zeng, Heehwa G. Son, Shadmehr Demehri

**Affiliations:** 1 Center for Cancer Immunology and Cutaneous Biology Research Center, Department of Dermatology and Krantz Family Center for Cancer Research, Department of Pathology, Massachusetts General Hospital and Harvard Medical School, Boston, Massachusetts, USA.; 2 Department of Urology and; 3 Guangdong Provincial Key Laboratory of Malignant Tumor Epigenetics and Gene Regulation, Guangdong-Hong Kong Joint Laboratory for RNA Medicine, Medical Research Center, Sun Yat-Sen Memorial Hospital, Sun Yat-Sen University, Guangzhou, China.

**Keywords:** Immunology, Oncology, Adaptive immunity, Breast cancer, Cytokines

## Abstract

CD4^+^ T cells contribute to antitumor immunity and are implicated in the efficacy of cancer immunotherapies. In particular, CD4^+^ T helper 2 (Th2) cells were recently found to block spontaneous breast carcinogenesis. However, the antitumor potential of Th2 cells in targeting established breast cancer remains uncertain. Herein, we demonstrate that Th2 cells induced by the topical calcipotriol/thymic stromal lymphopoietin cytokine axis suppressed the growth of established mammary tumors in mice. Interleukin-24 (IL-24), an anticancer cytokine, was highly upregulated in macrophages infiltrating calcipotriol-treated mammary tumors. Macrophages expressed IL-24 in response to IL-4 signaling in combination with Toll-like receptor 4 (TLR4) agonists (e.g., HMGB1) in vitro. Calcipotriol treatment significantly increased HMGB1 release by tumor cells in vivo. CD4^+^ T cell depletion reduced HMGB1 and IL-24 expression, reversing calcipotriol’s therapeutic efficacy. Macrophage depletion and TLR4 inhibition also reduced the therapeutic efficacy of calcipotriol. Importantly, calcipotriol treatment failed to control mammary tumors lacking the IL-24 receptor on tumor cells. Collectively, our findings reveal that Th2 cell–macrophage crosstalk leads to IL-24–mediated tumor cell death, highlighting a promising therapeutic strategy to tackle breast cancer.

## Abstract 2

We uncover the efficacy of topical calcipotriol for breast cancer therapy, which relies on inflammatory CD4^+^ Th2 cell-macrophage crosstalk to induce IL-24-mediated tumor cell death.

## Graphical abstract

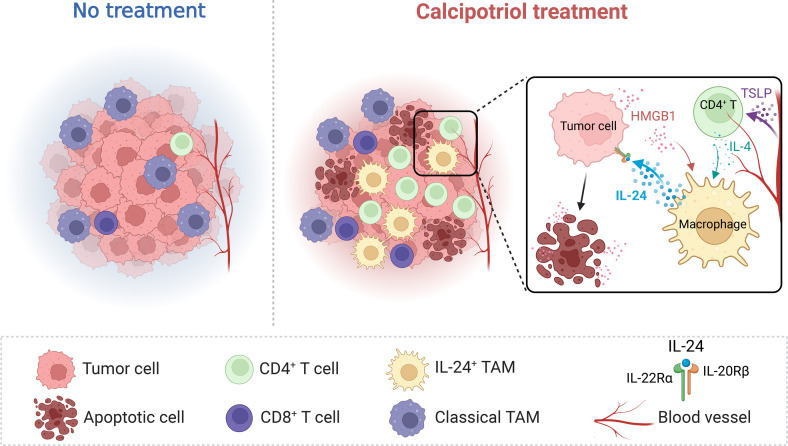

## Introduction

Breast cancer is a heterogeneous disease with low immunogenicity at baseline (1, 2). Although immune checkpoint blockade and adoptive T cell therapies have achieved therapeutic success in melanoma and other immunogenic cancers, these therapies have shown limited clinical benefit in breast cancer (3–6). Among CD4^+^ T cells implicated in cancer immunology, T helper 1 (Th1) cells and Tregs promote and suppress antitumor immunity in response to cancer immunotherapies, respectively (7, 8). Meanwhile, CD4^+^ Th2 cell–mediated immune response has traditionally been viewed as favoring tumor growth (9, 10). However, increasing evidence suggests that Th2 cell activation can result in direct or indirect cancer suppression (11–13). Devising a mechanism to activate the antitumor immune response through CD4^+^ Th2 cells may yield a significant therapeutic benefit in breast and other immunological cold cancers.

CD4^+^ T cells are important effector immune cells alongside CD8^+^ T cells in cancer (8, 14, 15). In bladder cancer, cytotoxic CD4^+^ T cells are clonally expanded and can kill autologous tumors in an MHC class II–dependent manner (15). We and others have previously demonstrated that epidermis-derived thymic stromal lymphopoietin (TSLP) creates robust antitumor response by activating CD4^+^ Th2 cells against early skin and breast carcinogenesis (11, 13). Importantly, skin-derived TSLP, which can be induced by an FDA-approved topical medication, calcipotriol, can reach and activate CD4^+^ T cell–mediated antitumor immunity in the tumor microenvironment (TME) (11, 13). Although TSLP-stimulated CD4^+^ Th2 cells are not cytotoxic themselves, they recruit a broad repertoire of immune effector cells to attack the cancer more effectively (11). Hence, identifying the key factors that are involved in CD4^+^ T cell–mediated tumor immunity in breast cancer will aid in boosting antitumor immunity against this disease.

Macrophages are a major target of CD4^+^ T cells in the TME (16–18). Th1 cells secret interferon-gamma (IFN-γ), which acts on macrophages to increase their phagocytosis and tumoricidal ability (18). In contrast, Th2 cells are the main cellular sources of interleukin 4 (IL-4) and IL-13, contributing to an alternative form of macrophage activation oriented toward tissue remodeling and immunoregulation (18, 19). Thus, tumor-associated macrophages (TAMs) comprise a continuum of phenotypes spanning from “antiinflammatory” to “proinflammatory,” which are governed by diverse mediators within the TME (20).

We have previously demonstrated that TSLP-stimulated CD4^+^ Th2 cell immunity suppresses early stages of breast cancer development (11, 13). To test the effect of CD4^+^ Th2 cell immunity in late-stage breast cancer, we used a cell line derived from a *MMTV-PyMt^tg^* mouse mammary tumor (the PyMt cell line) to establish mammary tumors in mice that represent late-stage breast cancer (11, 21, 22). We demonstrated that the induction of TSLP by topical calcipotriol treatment inhibited mammary tumor growth by activating CD4^+^ T cells. Interestingly, we discovered proapoptotic cytokine IL-24 expression by TAMs in response to TSLP-activated CD4+ Th2 cells in combination with Toll-like receptor 4 (TLR4) stimulation. Indeed, calcipotriol treatment showed markedly reduced efficacy in controlling Il20rb–/– PyMt tumor growth compared with WT PyMt tumor. Altogether, we identified a mechanism mediating Th2 cell immunity against established breast cancer growth, at least partly through TAMs that express IL-24, which leads to tumor suppression.

## Results

### Topical calcipotriol treatment suppresses mammary tumor growth.

**Figure 1:**
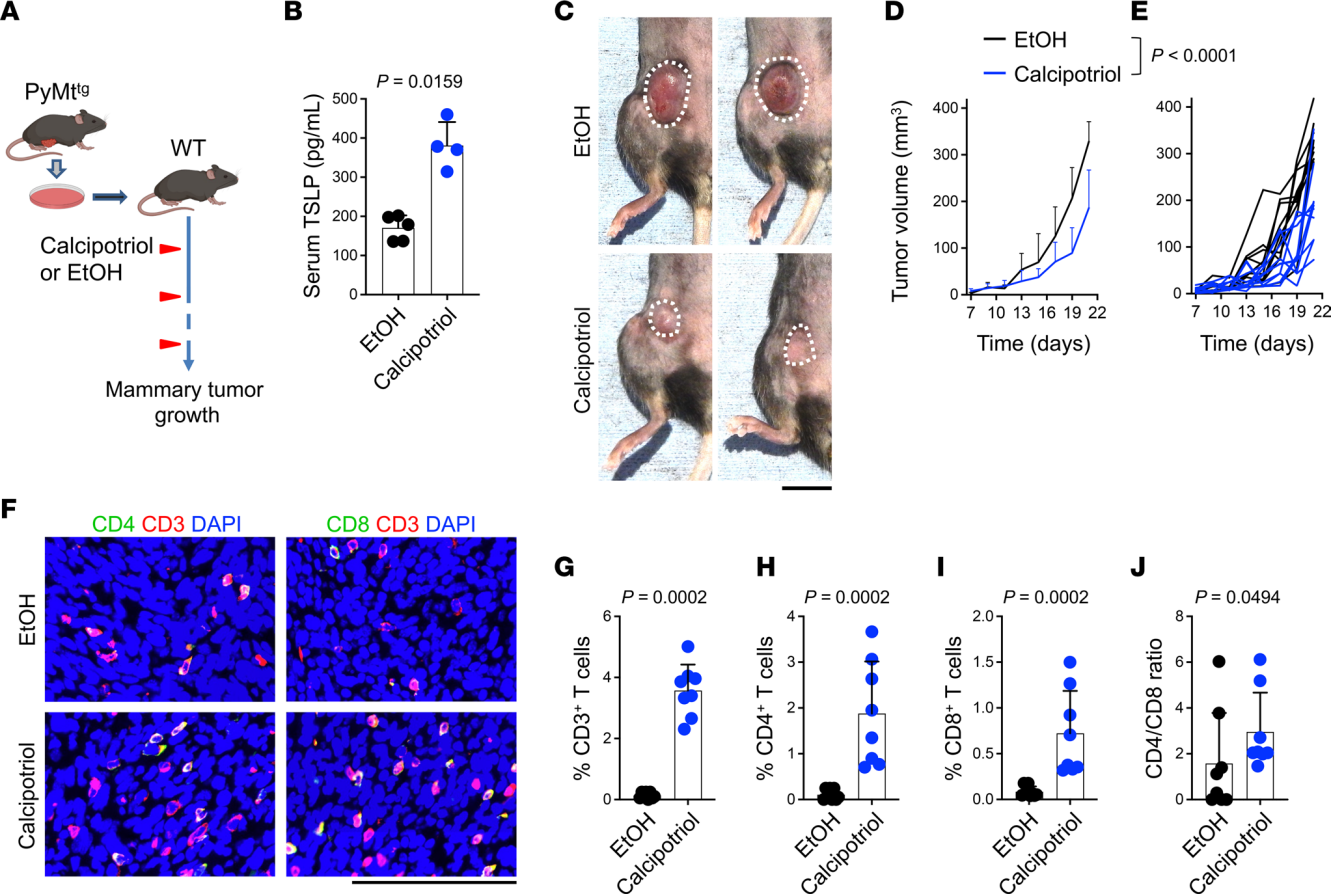
Topical calcipotriol treatment inhibits PyMt mammary tumor growth in WT mice. (**A**) Schematic diagram outlining the experimental design used to test the efficacy of topical calcipotriol treatment in blocking mammary tumor growth. Six- to eight-week-old WT mice were used as tumor recipients. The animals were treated with 20 nmol calcipotriol or EtOH topically every 2 days, starting at 2 days after orthotopic PyMt mammary tumor cell implantation. (**B**) TSLP levels in the circulation after topical calcipotriol versus EtOH treatment. (**C**–**E**) Topical calcipotriol treatment effect on PyMt mammary tumor growth shown as (**C**) representative macroscopic images of the mammary tumors at the endpoint (circles highlight the tumors), (**D**) mean tumor volumes + SD, and (**E**) spider plot of tumor volume over time (*n* = 10 in each group). (**F**–**J**) Tumor-infiltrating CD3^+^ T cells, CD4^+^ T cells, and CD8^+^ T cells shown as (**F**) representative immunofluorescence (IF) images of CD3-, CD4-, and CD8-stained tumor tissues; (**G**) percentage of CD3^+^ T cells of total cells; (**H**) percentage of CD4^+^ T cells of total cells; (**I**) percentage of CD8^+^ T cells of total cells; and (**J**) The ratio of CD4^+^ T to CD8^+^ T cells in calcipotriol- and EtOH-treated mammary tumors (*n* = 8 in each group). Bar graphs show mean + SD. Mann-Whitney *U* test (**B** and **G**–**J**) and 2-way ANOVA (**D** and **E**). Scale bars: 1 cm (**C**); 100 μm (**F**).

Calcipotriol induces TSLP expression, leading to Th2 cell activation during early carcinogenesis (11, 13). In cell line–based breast cancer models, “late-stage” refers to models that rapidly progress and metastasize, mimicking advanced breast cancer, contrasting with spontaneous models, in which tumor growth follows a more natural, variable timeline (23). To elucidate the effect of TSLP/Th2 cell induction by calcipotriol on late-stage breast cancer progression, we used a PyMt cell line and established an orthotopic PyMt breast tumor model (11). We administered 20 nmol calcipotriol or EtOH solvent topically over the tumor site every 2 days, starting at 2 days after PyMt mammary tumor cell implantation into WT mice (Figure 1A). Topical calcipotriol treatment led to a significant increase in circulating TSLP levels (Figure 1B). The expression of TSLP in serum gradually decreased to baseline after 5 days following the cessation of topical treatment (Supplemental Figure 1; supplemental material available online with this article; https://doi.org/10.1172/jci.insight.180962DS1). WT animals that received calcipotriol developed significantly smaller tumors compared with those of EtOH-treated controls (*P* < 0.0001, Figure 1, C–E). A markedly higher number of CD3^+^ T, CD4^+^ T, and CD8^+^ T cells infiltrated the calcipotriol- compared with EtOH-treated tumors (Figure 1, F–I). Importantly, the CD4^+^ T/CD8^+^ T cell ratio was significantly increased in calcipotriol- compared with EtOH-treated tumors, demonstrating that CD4^+^ T cells constituted the majority of tumor-infiltrating T cells in calcipotriol-treated PyMt mammary tumors (Figure 1, H–J). Thus, calcipotriol exhibits a robust antitumor effect in late-stage breast cancer associated with TSLP induction and CD4^+^ T cell infiltration into the tumors.

### Calcipotriol upregulates Il24 expression in mammary tumors.

**Figure 2:**
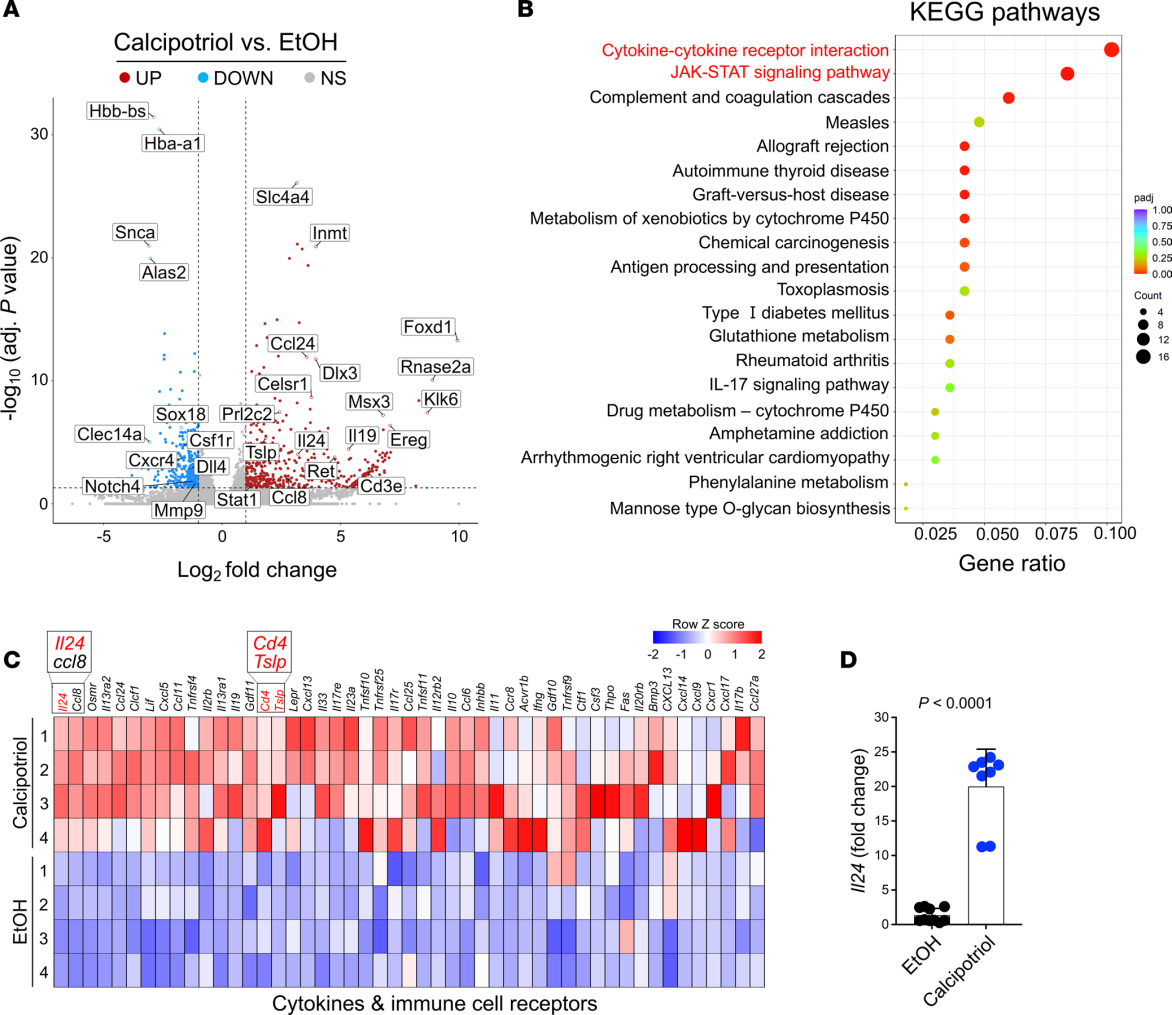
Calcipotriol treatment induces *Il24* expression in mammary tumors. (**A**) Volcano plot illustrating the differential gene expression between calcipotriol- and EtOH-treated PyMt tumors. Genes up- and downregulated in calcipotriol-treated tumors are highlighted in red and blue, respectively. (**B**) KEGG pathway analysis of RNA-Seq results, highlighting pathways enriched in calcipotriol- compared with EtOH-treated mammary tumors. (**C**) Heatmap of differential gene expression associated with the cytokine-cytokine receptor interaction in calcipotriol versus EtOH treatments. (**D**) qRT-PCR gene expression analysis for *Il24* across calcipotriol- and EtOH-treated PyMt tumors (*n* = 8 in each group). Results are depicted as fold change relative to the EtOH group. Bar graph shows mean + SD. Mann-Whitney *U* test (**D**).

To determine the mechanism involved in mediating the tumor-suppressing effect of calcipotriol, we performed RNA-Seq on PyMt mammary tumors 24 hours after the last topical treatment of calcipotriol versus EtOH. Genes associated with inflammation and JAK/STAT signaling were significantly upregulated, and genes associated with angiogenesis and tissue remodeling were downregulated in tumors treated with calcipotriol (Figure 2A). KEGG pathway analysis also showed that cytokine-cytokine receptor interaction pathway was significantly upregulated in calcipotriol-treated tumors compared with EtOH-treated tumors (Figure 2B). Notably, *Il24*, an antitumor gene (24), was the most significantly upregulated gene in the cytokine and cytokine receptors gene set (Figure 2C). The upregulation of *Il24* expression in calcipotriol-treated tumors was validated by quantitative real-time PCR (qRT-PCR) (Figure 2D). Likewise, *Tslp* and macrophage chemotactic factor *ccl8* were significantly upregulated in calcipotriol-treated tumors compared with EtOH-treated tumors (Figure 2C and Supplemental Figure 2). These findings suggest that IL-24 may play an important role in mediating the antitumor efficacy of calcipotriol therapy in late-stage breast cancer.

### Calcipotriol’s antitumor effect and IL-24 induction depend on TSLP and CD4^+^ T cells.

**Figure 3:**
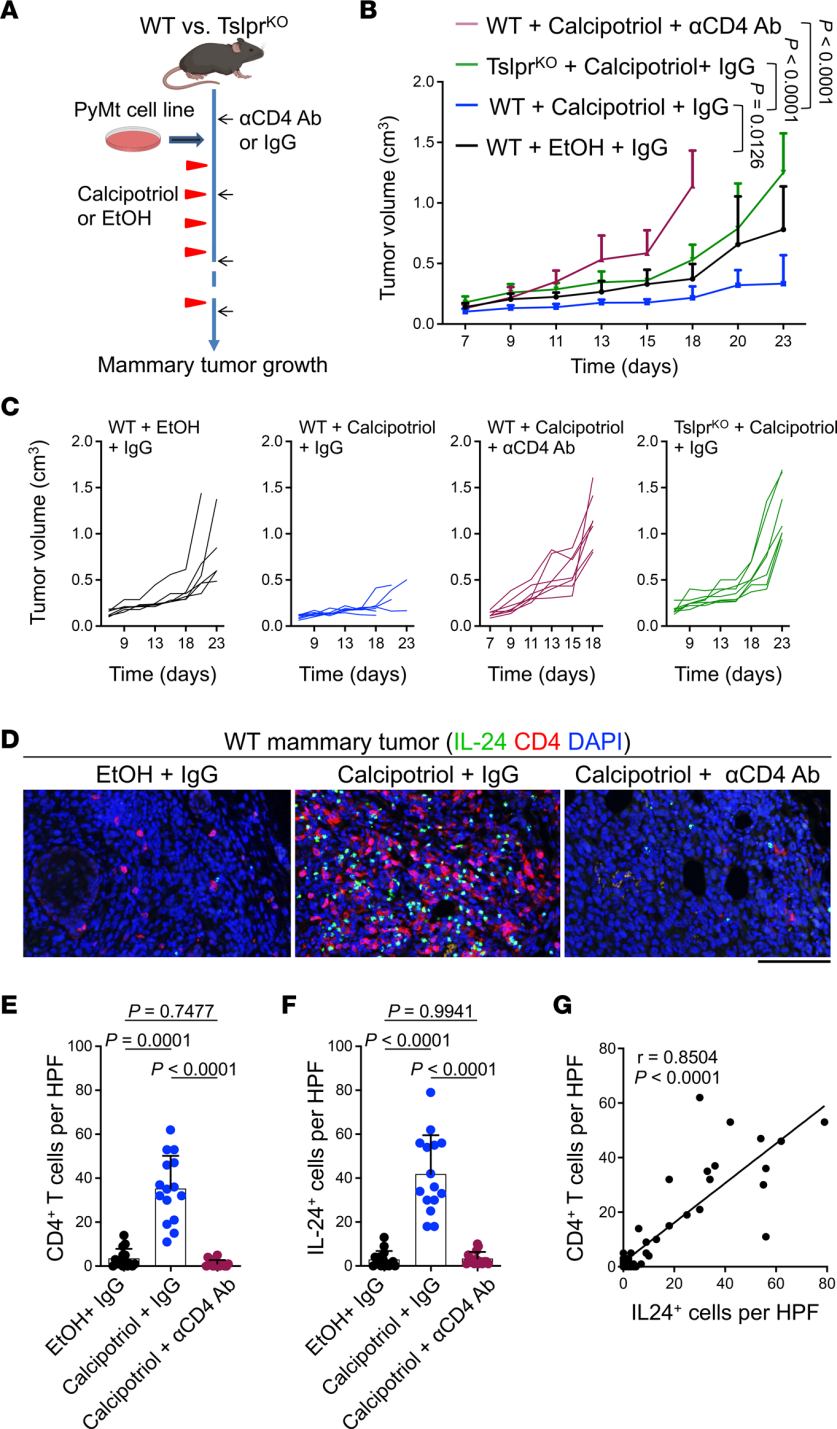
Calcipotriol-mediated mammary tumor suppression and IL-24 induction rely on TSLP and CD4^+^ T cells. (**A**) Schematic diagram outlining the experimental approach to assess the mechanism of topical calcipotriol treatment in inhibiting mammary tumor growth. WT and Tslpr^KO^ mice served as tumor recipients, which were treated with anti-CD4 Ab (αCD4 Ab) or IgG control every 5 days starting 1 day before tumor cell implantation. Two days after orthotopic PyMt mammary tumor cell implantation, mice were topically treated with 20 nmol calcipotriol or EtOH every 2 days. (**B** and **C**) PyMt mammary tumor growth in WT and Tslpr^KO^ mice subjected to calcipotriol versus EtOH treatments, with or without CD4^+^ T cell depletion, shown as (**B**) mean tumor volumes + SD and (**C**) spider plot. WT + EtOH + IgG (*n* = 6), WT + calcipotriol + IgG (*n* = 7), WT + calcipotriol + αCD4 Ab (*n* = 7), and Tslpr^KO^ + calcipotriol + IgG (*n* = 7). (**D**) Representative IF images of mammary tumors stained for CD4 and IL-24. (**E** and **F**) Quantification of (**E**) CD4^+^ T cells and (**F**) IL-24^+^ cells in WT mammary tumors treated with EtOH + IgG, calcipotriol + IgG, or calcipotriol + αCD4 Ab. Each dot represents a high-power field (HPF) image. Three HPF images from 5 tumors are included in each group. (**G**) Correlation between the presence of CD4^+^ T cells and IL-24^+^ cells in WT mammary tumors. Bar graphs show mean + SD, 1-way ANOVA (**E** and **F**), 2-way ANOVA (**B** and **C**), and Spearman’s rank correlation analysis (**G**). Scale bar: 100 μm (**D**).

We investigated whether the ability of calcipotriol to suppress PyMt mammary tumor growth relied on TSLP and CD4^+^ T cells. We compared PyMt mammary tumor growth in WT and *Tslpr^–/–^* (Tslpr^KO^) mice treated with 20 nmol calcipotriol or EtOH topically every 2 days, starting at 2 days after tumor cell implantation (Figure 3A). To determine the role of CD4^+^ T cells in calcipotriol-mediated antitumor effects, we also injected a subgroup of calcipotriol-treated WT mice with anti-CD4 Ab starting 1 day before tumor cell implantation (Figure 3A). As expected, WT animals that received calcipotriol developed significantly smaller tumors compared with EtOH-treated WT controls (*P* = 0.0126, Figure 3, B and C). However, Tslpr^KO^ mice treated with calcipotriol developed significantly larger tumors than calcipotriol-treated WT controls (*P* < 0.0001, Figure 3, B and C). Moreover, CD4^+^ T cell–depleted calcipotriol-treated WT mice showed a significant acceleration of tumor growth compared with calcipotriol-treated WT mice (*P* < 0.0001, Figure 3, B and C).

To further evaluate whether IL-24 induction by calcipotriol was linked to CD4^+^ T cell infiltration in mammary tumors, we assessed the colocalization of IL-24–expressing cells with CD4^+^ T cells in PyMt tumors of WT mice treated with EtOH, calcipotriol, or calcipotriol plus anti-CD4 Ab. The number of IL-24^+^ cells was significantly increased in calcipotriol- compared with EtOH-treated tumors, but it was markedly decreased upon CD4^+^ T cell depletion in calcipotriol-treated tumors (*P* < 0.0001, Figure 3, D–F). In addition, we observed a strong correlation between IL-24^+^ cells and CD4^+^ T cell infiltration in tumors across all 3 groups (*r* = 0.8504, *P* < 0.0001, Figure 3G). Notably, IL-24 did not colocalize with CD4 in the same cells, indicating that IL-24 was not expressed by CD4^+^ T cells in calcipotriol-treated tumors (Figure 3D). *Il24* expression was significantly lower in calcipotriol-treated tumors of Tslpr^KO^ mice compared with those of WT controls (Supplemental Figure 3). Collectively, these results demonstrate that CD4^+^ T cells and TSLP signaling are required for calcipotriol-induced antitumor response against late-stage breast cancer, which is accompanied by a significant accumulation of IL-24^+^ cells within the TME.

### Calcipotriol treatment leads to IL-24 expression by TAMs in mammary tumors.

**Figure 4:**
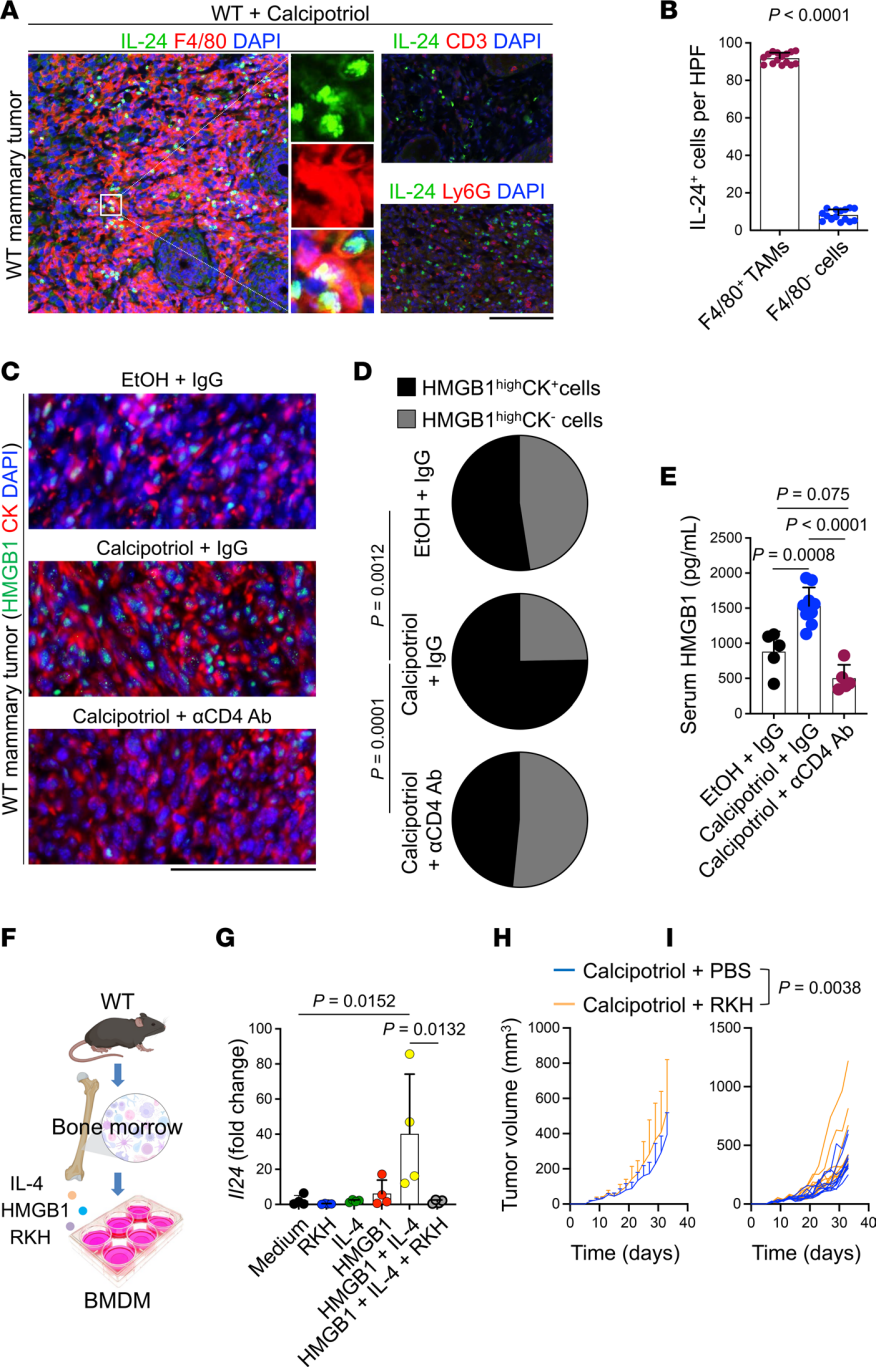
IL-24 is mainly expressed by TAMs in mammary tumors treated with calcipotriol. (**A**) Representative IF images of IL-24–, F4/80-, Ly6G-, and CD3-stained mammary tumor in WT mice treated with topical calcipotriol. (**B**) Quantification of IL-24^+^ F4/80^+^ TAMs versus IL-24^+^ F4/80^-^ cells in PyMt mammary tumors treated with calcipotriol. Each dot represents a high-power field (HPF) image. Five HPF images from 3 tumors are included in each group. (**C**) Representative images of HMGB1- and cytokeratin-stained (CK-stained) PyMt mammary tumor in WT mice treated with EtOH + IgG, calcipotriol + IgG, or calcipotriol + αCD4 Ab. (**D**) Distribution of HMGB1^hi^-expressing cells in CK^–^ and CK^+^ cells in PyMt mammary tumors of WT mice treated with EtOH + IgG (*n* = 6), calcipotriol + IgG (*n* = 7), or calcipotriol + αCD4 Ab (*n* = 7). (**E**) The serum HMGB1 levels in PyMt mammary tumor-bearing WT mice treated with EtOH + IgG (*n* = 5), calcipotriol + IgG (*n* = 9), or calcipotriol + αCD4 Ab (*n* = 5). (**F**) Schematic diagram outlining the experimental design used to test *Il24* induction in BMDMs generated from WT mice stimulated with IL-4, HMGB1, and a TLR4 antagonist, RKH, alone or in combination. (**G**) *Il24* mRNA levels 3 hours after exposure to the indicated stimulations (*n* = 4 in each group). (**H** and **I**) PyMt mammary tumor volume in WT animals treated by topical calcipotriol with PBS (control) versus RKH. 150 mg/kg RKH was injected intraperitoneally into mice twice a week, starting a day before tumor inoculation. Data are presented as (**H**) mean tumor volumes + SD and (**I**) spider plot (*n* = 10 in each group). Bar graphs show mean + SD, Mann-Whitney *U* test (**B**), 1-way ANOVA (**E** and **G**), Fisher’s exact test (**D**), and 2-way ANOVA (**H** and **I**). Scale bars: 100 μm (**A** and **C**).

To determine the cellular source of IL-24 in the TME, we investigated IL-24 colocalization with pan-macrophage marker, F4/80; neutrophil marker, Ly6G; and T cell marker, CD3 (Figure 4A). IL-24 was predominantly colocalized with F4/80^+^ macrophages but not neutrophils or T cells in the TME (Figure 4, A and B). Notably, the number of F4/80^+^ macrophages significantly increased in calcipotriol-treated tumors, and macrophage depletion reduced the efficacy of calcipotriol treatment (Supplemental Figure 4). Interestingly, while CD4^+^ T cell depletion in calcipotriol-treated tumors reduced IL-24^+^ macrophages, it had no significant impact on the total number of macrophages in the tumor (Figure 3, D–F, and Supplemental Figure 4, A and B). These findings suggest that calcipotriol-activated CD4^+^ T cells are not responsible for promoting macrophage tumor infiltration but instead play a critical role in IL-24 production by macrophages within the TME.

Next, we explored whether TSLP and/or the CD4^+^ Th2-associated cytokine IL-4 could trigger IL-24 expression in macrophages. Although neither TSLP nor IL-4 alone was sufficient to induce IL-24 expression, the combination of IL-4 and the TLR4 agonist, LPS, increased *Il24* levels in bone marrow–derived macrophages (BMDMs) (Supplemental Figure 5). Interestingly, we discovered that high-mobility group box 1 (HMGB1), which also signals through TLR4 (25), was highly expressed in mammary tumor cells and elevated in the sera of calcipotriol-treated mice compared with ethanol-treated and CD4^+^ T cell–depleted mice (Figure 4, C–E). Purified HMGB1 combined with IL-4 markedly increased *Il24* expression in BMDMs (Figure 4, F and G). To confirm the involvement of TLR4 in this process, we used a TLR4 antagonist, Arg-Lys-His (RKH) (26), in the culture system, which successfully reversed HMGB1 plus IL-4–induced Il24 expression in BMDMs. Additionally, to further validate TLR4’s role in the therapeutic effects of calcipotriol, we administered RKH to PyMt tumor-bearing mice during calcipotriol treatment. RKH significantly impaired the therapeutic efficacy of calcipotriol (Figure 4, H and I). These findings indicate that TAMs express IL-24 in calcipotriol-treated mammary tumors triggered by type 2 cytokines plus TLR4 activation.

### The IL-24/IL-20R axis is required for the antitumor effect of calcipotriol treatment in mammary tumors.

**Figure 5:**
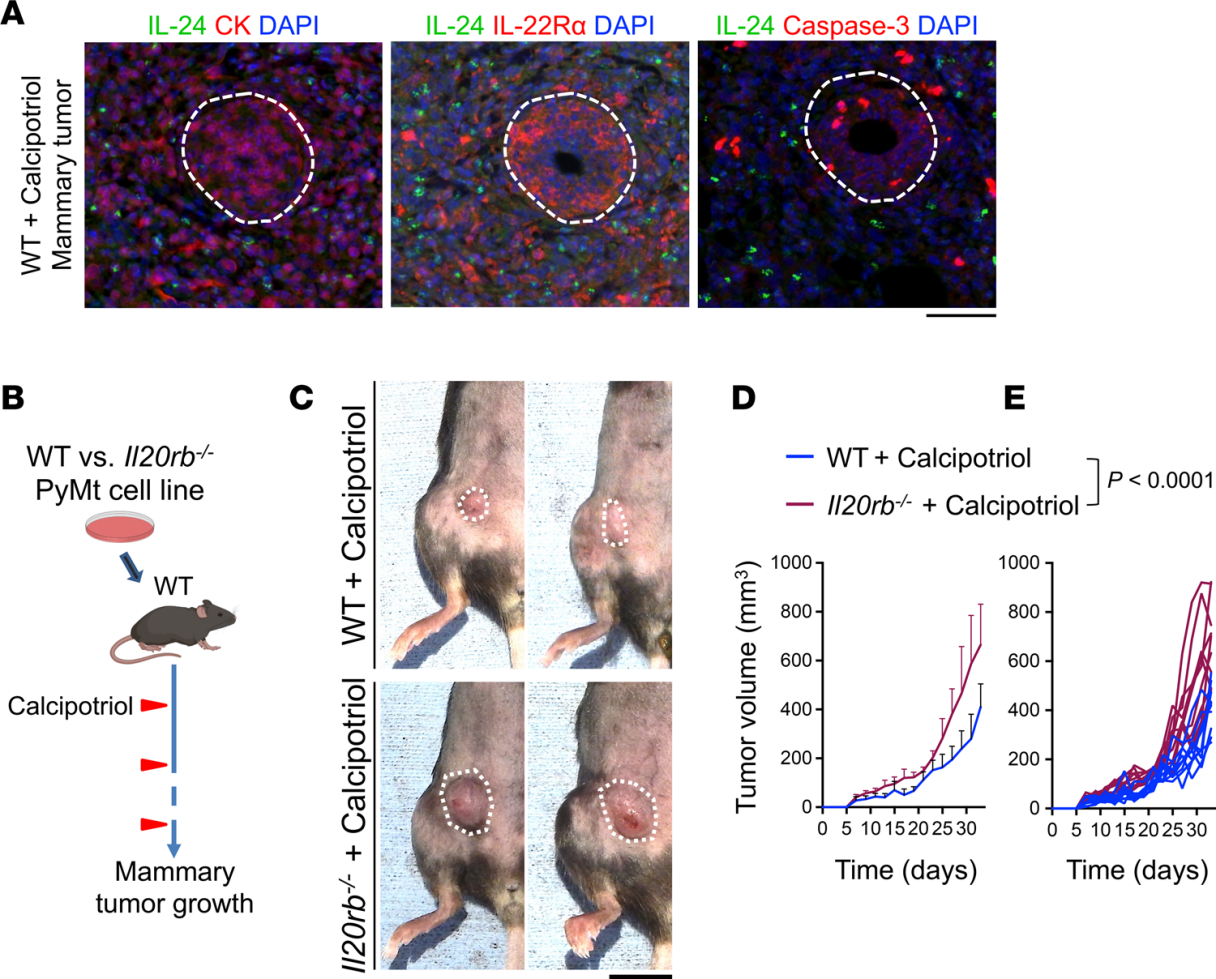
Deletion of the IL-24 receptor in tumor cells blocks the antitumor effect of calcipotriol on mammary tumors. (**A**) Representative IF images of IL-24, IL-22Rα, and cleaved caspase-3–stained PyMt mammary tumor in calcipotriol-treated WT mice. Dotted circles outline a tumor focus. (**B**) Schematic diagram of the experimental setup to assess the contribution of the IL-24/IL-20R axis to the efficacy of topical calcipotriol treatment against PyMt mammary tumor growth. (**C**) Representative macroscopic images of WT and *Il20rb^–/–^* PyMt mammary tumors in WT mice (dotted circles highlight the tumor sites). (**D** and **E**) WT versus *Il20rb^–/–^* PyMt mammary tumor volume over time in calcipotriol-treated WT mice presented as (**D**) mean tumor volumes + SD and (**E**) spider plot (*n* = 10 in each group). Two-way ANOVA (**D** and **E**). Scale bars: 100 μm (**A**) and 1 cm (**C**).

IL-24 binding to IL-20 receptor (IL-20R, composed of IL-20α/IL-22Rα and IL-20Rβ subunits) activates downstream signaling cascades leading to tumor cell apoptosis (27). To determine whether the IL-24/IL-20R axis contributed to calcipotriol-mediated mammary tumor suppression, we first examined the association between IL-24 induction by calcipotriol in the TME and the tumor cell expression of IL-22Rα and apoptosis marker, cleaved caspase-3. In the TME, cleaved caspase-3 colocalized with IL-22Rα–expressing tumor cells after calcipotriol treatment (Figure 5A). Next, we generated the *Il20rb^–/–^* PyMt mammary tumor cell line using CRISPR/Cas9 gene editing technology (Supplemental Figure 6A). *Il20rb^–/–^* PyMt mammary tumor cells showed a similar proliferation rate to WT PyMt cells in vitro (Supplemental Figure 6B). WT or *Il20rb^–/–^* PyMt mammary tumors were implanted in WT mice and treated with 20 nmol calcipotriol topically every 2 days, starting at 2 days after tumor cell implantation (Figure 5B). Calcipotriol-treated *Il20rb^–/–^* PyMt cells developed significantly larger tumors compared with calcipotriol-treated WT PyMt cells (*P* < 0.0001, Figure 5, C–E). By contrast, EtOH-treated *Il20rb^–/–^* PyMt tumors showed tumor growth kinetics similar to those of EtOH-treated WT PyMt tumors (Supplemental Figure 6, C–F). Thus, the IL-24/IL-20R axis plays an essential role in mediating the efficacy of calcipotriol in suppressing mammary tumor growth.

### IL-24 expression is associated with improved prognosis in human breast cancer.

**Figure 6:**
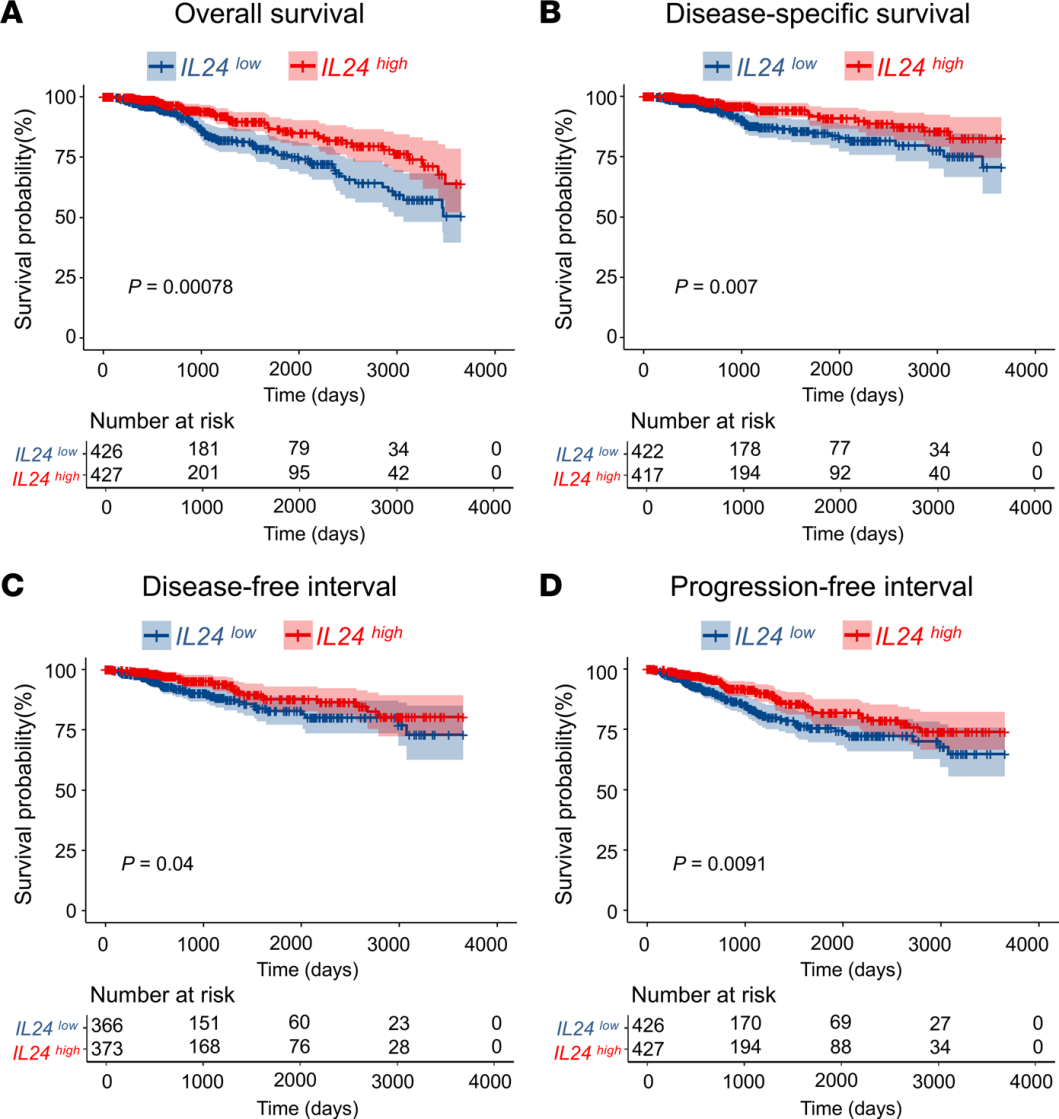
Correlation of *IL24* mRNA expression with clinical outcomes in patients with breast cancer. Kaplan-Meier survival curves derived from TCGA BRCA cohort, categorizing patients based on *IL24* expression. Patients were segmented into high- and low-expression groups using the 40% *IL24* expression threshold. Outcomes are delineated as (**A**) overall survival, (B) disease-specific survival, (**C**) disease-free interval, and (**D**) progression-free interval. Log-rank test (**A**–**D**).

To assess the effect of IL-24 expression on human breast cancer, we interrogated The Cancer Genome Atlas (TCGA) database for the association between *IL24* expression and survival in the breast cancer cohort. By categorizing patients into groups based on their *IL24* expression, distinguishing between those with low and high expression levels using a fortieth percentile threshold, high *IL24* expression was significantly associated with improved overall survival (*P* = 0.00078, Figure 6A). The 10-year survival rate of patients with high *IL24* expression was 64% as compared with 51% in patients with low *IL24* expression. Clinicopathological indicators (age, tumor stage, nodal stage, metastasis stage) predicted poorer overall survival in those with low IL24 expression compared with those with high IL24 expression in the univariate analysis (Supplemental Table 1). The clinicopathological variables that were significant in univariate analysis were included as covariates. Multivariate analysis revealed that *IL24* expression was still an independent predictor for overall survival (HR = 0.651, *P* = 0.043). Moreover, *IL24* expression was associated with improved disease-specific survival (*P* = 0.007, Figure 6B), disease-free interval (*P* = 0.04, Figure 6C), and progression-free interval (*P* = 0.00091, Figure 6D). Thus, IL-24 upregulation may play a protective role in human breast cancer.

## Discussion

Our findings reveal that TSLP-stimulated CD4^+^ Th2 cells suppress mammary tumor growth by promoting IL-24^+^ TAM development and IL-24–mediated tumor cell apoptosis. Consistent with the dominant role of CD4^+^ Th2 cells in mediating TSLP’s antitumor effects during early carcinogenesis (11, 13), topical TSLP induction by calcipotriol treatment results in the induction of CD4^+^ Th2 cell immunity against late-stage breast cancer progression. This immune response is distinct from the Th1/CD8^+^ T cell immunity commonly found to mediate the efficacy of existing cancer immunotherapies (28). Interestingly, IL-24 is the most significantly upregulated cytokine in calcipotriol-treated mammary tumors (Figure 2, B and C). IL-24 is expressed by TAMs, and its expression is TSLP and CD4^+^ T cell dependent. Although TSLP and IL-4 alone failed to induce IL-24 expression, a combination of IL-4 with TLR4 agonists elevated IL-24 levels in BMDMs in vitro. Functional studies demonstrate that IL-24 signaling through its receptor, IL-20R, is required to induce mammary tumor suppression. Thus, CD4^+^ Th2 cell activation can suppress breast cancer by inducing antitumor IL-24–expressing TAMs in the TME, which can be leveraged in cancer immunotherapy.

Topical calcipotriol is a potent inducer of TSLP expression by keratinocytes, which drives type 2 immune response in mice and humans (11, 29). Studies on breast cancer cell line models have shown that the endogenous TSLP signal is co-opted by cancer cells to promote their growth (11, 30). In particular, myeloid cell–derived TSLP promotes breast cancer cell survival through the induction of an antiapoptotic molecule, Bcl-2 (31). By contrast, TSLP induction stimulates the specific expansion and antitumor functionality of CD4^+^ Th2 cells in spontaneous and cell line–derived breast tumors (11, 13). Calcipotriol-induced TSLP released from skin keratinocytes primarily leads to the activation of Th2 cells that drive breast cancer cells into terminal differentiation during early-stage tumor development (11, 13). Our present findings demonstrate that calcipotriol-induced TSLP also leads to the activation of CD4^+^ Th2 cells that promote tumor suppression during late-stage tumor progression. Thus, calcipotriol and other agents that can induce TSLP will trigger CD4^+^ Th2 immunity for early and late breast cancer treatment.

Cytokines play important roles in mediating the pro- and antitumor effects of CD4^+^ T cells (32). CD4^+^ Th1 cells secrete IFN-γ and TNF-α to trigger CD8^+^ T and NK cells to eliminate tumor cells (15, 33). Th2 cells that promote tumor development exhibit a regulatory profile associated with high levels of IL-10 and TGF-β secretion (34). In contrast, inflammatory Th2 cells activated by TSLP express high levels of IL-3, IL-5, and GM-CSF that can effectively protect the breast (35) against oncogene-driven malignant transformation (13). Furthermore, TSLP-activated Th2 cells express high levels of TNF-α that can suppress spontaneous breast carcinogenesis (13). In this study, we further showed that calcipotriol-induced TSLP activates Th2 cells to release IL-4, which in turn promotes IL-24 production by TAMs, resulting in tumor cell apoptosis. By contrast, in a 4T1 breast tumor model, baseline tumor cell–derived TSLP promotes the survival of the tumor cells through induction of the expression of antiapoptotic molecule, Bcl-2 (31). The distinct effects of TSLP observed in the two studies may reflect that baseline TSLP engages an innate immune axis in the tumor, while TSLP induction by calcipotriol treatment leads to robust antitumor adaptive immunity.

IL-24, initially identified as melanoma differentiation-associated gene-7 or MDA-7, is an anticancer cytokine within the IL-10 gene family (36–39). IL-24’s anticancer function as a cytokine involves its interaction with the IL-20R, leading to endoplasmic reticulum stress–induced tumor cell apoptosis (27). Activated macrophages’ release of IL-24 can inhibit tumor cell growth without harming normal cells (36–43). In breast cancer, IL-24 exerts an inhibitory effect on breast tumor development (27, 42, 44–46). In these studies, IL-24 overexpression has been used to elucidate its anticancer function (24, 44, 45, 47). It is important to identify endogenous sources of IL-24 to further characterize its effects in vivo. IL-24 can be expressed by melanocytes, keratinocytes, and immune cells, including T cells and monocytes/macrophages (40, 47). We found that macrophages increased in calcipotriol-treated tumors independent of CD4^+^ T cell induction, which may be mediated by CCL8 upregulation in the TME. However, IL-24–expressing macrophages are increased in a CD4^+^ T cell–dependent manner after calcipotriol treatment, pointing to type 2 cytokine release by Th2 cells as well as TLR4 stimulation as necessary components for IL-24 induction in macrophages (40). We also found that HMGB1, a TLR4 agonist that can induce IL-24, is highly upregulated in calcipotriol-treated tumor cells in a CD4^+^ T cell–dependent manner, suggesting that it may contribute to IL-24 induction in macrophages.

HMGB1 is a highly conserved DNA-binding protein, which has paradoxical biological functions inside as well as outside the cell. While HMGB1 dysfunction can contribute to tumorigenesis (25), intracellular HMGB1 in breast cancer can function as a tumor suppressor by inducing cell cycle arrest and apoptosis (48). The extracellular HMGB1 activates the TLR4 complex (25). We showed that HMGB1 plus IL-4 promotes IL-24 production by macrophages in a TLR4-dependent manner. Although the regulation and function of IL-24+ TAMs need further investigation to be elucidated, our data suggest that they play an essential role in CD4^+^ Th2 cell–mediated antitumor immune response.

In summary, we demonstrate the efficacy of topical calcipotriol in inhibiting mammary tumor growth in a TSLP- and CD4^+^ T cell–dependent manner. Th2 cell–derived cytokines upregulate anticancer IL-24 cytokine expression by TAMs to kill tumor cells. Thus, calcipotriol treatment can be a promising therapeutic treatment for breast cancer by triggering CD4^+^ T cell–macrophage crosstalk to induce antitumor immunity.

## Methods

### Sex as a biological variable.

In our study, all the mice used were female, as breast cancer predominantly occurs in female individuals.

### Mice.

WT female mice on the C57BL/6 background were purchased from Charles River Laboratories Inc. Tslpr^KO^ mice (a gift of Warren J. Leonard, NIH, Bethesda, Maryland) were maintained on the C57BL/6 background. All mice were housed under specific pathogen–free conditions with a 12-hour-light/dark cycle in the animal facility at Massachusetts General Hospital in compliance with animal care and all other relevant regulations.

### Cell line.

The PyMt mammary tumor cell line used in this study was derived from a mammary tumor of an MMTV-PyMt transgenic mouse on the C57BL/6 background, which was provided by Dr. David G. DeNardo at Washington University, St. Louis, Missouri, USA (11). Cells were maintained in DMEM (Cytiva, SH30022) containing high glucose and 10% heat-inactivated FBS supplemented with nonessential amino acids (Thermo Fisher Scientific, 11-140-050), 1 mmol/L sodium pyruvate (Cytiva, SH30239.01), 2 mmol/L glutamine (Cytiva, SH30228), and 100 units/mL penicillin/ streptomycin (Caisson Labs, PSL02) at 37°C and 5% CO_2_. Cells were screened for mouse Ab production and mycoplasma yearly. We used 6–8 passages for this study. Guide RNA (gRNA) sequences used were as follows: nonsense control, GCGACATCCTCATCTCGTTAGTA; *Il20rb* gRNA, GGGAAGCCTCACCGCGGCGCC. Nonsense control and gRNAs against *Il20rb* were cloned into the 293T cells and transfected with LentiCRISPRv2Cre (Addgene, 82415), D8.2 (Addgene, 8455), and VSV-G (Addgene, 8454) plasmids in a 4:3:1 ratio using lipofectamine 2000 (Thermo Fisher Scientific, 11668019) using the manufacturer’s instructions. To build the stable PyMt transfectant, lentivirus packaged from 293T cells was transfected into PyMt cells for 48 hours, along with polybrene (EMD Millipore, TR-1003); GFP^+^ cells were sorted using SH800S sorter (Sony).

### Orthotopic mammary tumor implantation and calcipotriol treatment.

Cells were passaged at 70% confluency, and 1 × 10^5^ PyMt-expressing nonsense control or *Il20rb* gRNA mammary tumor cells were orthotopically injected into the inguinal mammary fat pad of WT or Tslpr^KO^ mice under isoflurane anesthesia. For calcipotriol treatment, experimental mouse PyMt mammary tumors were treated topically with 20 nmol calcipotriol (Sigma-Aldrich, C4369-10MG) dissolved in 100% ethanol every 2 days, starting 2 days after tumor transfer. In each experiment, all animals in the test and control groups were treated with the same dose of topical calcipotriol. Tumor onset and tumor growth were monitored every 2–3 days. For macrophage depletion, 50 μL clodronate liposome or a control liposome (Liposoma, CP-010-010) was administered intraperitoneally starting 1 day prior to tumor cell injection and every week after tumor cell injection. The efficiency of F4/80^+^ macrophage depletion was assessed by flow cytometry. For inhibition of the TLR4 signaling pathway, 150 mg/kg TLR4-specific antagonist RKH or control PBS was administered intraperitoneally starting 1 day before tumor cell injection and twice a week after tumor cell injection. Tumors were harvested 24 hours after the last calcipotriol treatment. Tumor volume was measured and calculated using the formula (volume = length [mm] × width [mm]^2^/2).

### CD4^+^ T cell depletion.

1 × 10^5^ PyMt mammary tumor cells were injected orthotopically into the left inguinal mammary fat pad of mice. For CD4^+^ T cell depletion, 200 mg anti-CD4 (BioXCell, clone GK1.5, BE0003) or an IgG isotype control Ab (Southern Biotech, 0127-01) diluted in 200 mL sterile PBS was administered intraperitoneally starting 1 day before tumor cell injection and every 5 days after tumor cell injection. The efficiency of CD4^+^ T cell depletion was assessed by immunohistochemistry.

### Immunofluorescence staining.

Formalin-fixed, paraffin-embedded tumor samples were cut into 5 μm sections, which were processed for immunofluorescence as previously described (13, 49). Briefly, slides were immersed in an antigen unmasking solution (Vector Laboratories) at a 1:100 dilution in distilled water. Antigen retrieval was then performed in an electric high-pressure cooker (Cuisinart) for approximately 20 minutes. Unspecific protein binding sites were blocked with 5% BSA (Thermo Fisher Scientific) for 1 hour at room temperature. Sections were then incubated with mouse-specific anti–IL-24 (R&D Systems, MAB2786), anti-CD3 (Abcam, ab11089), anti-CD4 (Abcam, ab183685), anti-CD8 (CST, 98941S), anti-F4/80 (CST, 70076S), anti-Ly6G (CST, 31469S), anti-HMGB1 (CST, 6893S), and cytokeratin (Dako, M3515), followed by fluorochrome-conjugated secondary Abs (Supplemental Table 2). Sections were counterstained with DAPI nuclear stain (Thermo Fisher Scientific). Slides were scanned using the NanoZoomer s60 digital scanner (Hamamatsu Corp.), and high-resolution images were acquired using a Zeiss Axio Observer Z1 and analyzed using the Zeiss ZEN Image Processing software. Quantification of cell population and number was performed with HALO Image Analysis Platform (Indica Labs).

### Generations of BMDMs.

To generate BMDMs, bone marrow cells were collected from femurs and tibias of 6- to 8-week-old WT C57BL/6 mice (50). After erythrocytes were lysed using RBC Lysis Buffer (Biolegend, 420301), 1 × 10^6^/mL cells were seeded into 6-well plates. BMDM culture medium required DMEM containing high glucose and 10% heat-inactivated FBS supplemented with nonessential amino acids, 1 mmol/L sodium pyruvate, 2 mmol/L glutamine, 100 units/mL penicillin/streptomycin, and 25 ng/mL murine M-CSF (BioLegend, 576402). All media were substituted with fresh culture medium every 3 days. At day 6–7, BMDM purity was confirmed by using a Fortessa LSRII flow cytometer (BD Bioscience).

### BMDM stimulation with IL-4, TLSP, LPS, HMGB1, and RTH.

To test the expression of *Il24* by different stimulation, 1 × 10^6^/mL cultured BMDMs were stimulated with 10 ng/mL IL-4 (BioLegend, 574002), 10 ng/mL TSLP (BioLegend, 582402), 100 ng/mL LPS (Sigma-Aldrich, L6529), 500 ng/mL HMGB1 (Leadgene Biomedical, LDG002PME), or 500 μM RKH (MedChemExpress LLC, HY-P10208) alone or in combination for 3 hours. Gene expression of *Il24* was quantified by qRT-PCR.

### RNA isolation.

Methods for tumor RNA were extracted as previously reported (13). For mammary tumor RNA extraction, mammary tumor samples were homogenized in RLT buffer (Qiagen, 79216) supplemented with 1% 2-mercaptoethanol (Thermo Fisher Scientific, 21985-023) using a TissueLyser II (Qiagen) at a frequency of 30/s for 5 minutes. Samples were further lysed by resuspending the homogenized tissue in TRIzol Reagent (Thermo Fisher Scientific, 15-596-018) and stored at –80°C until use. For culture cell RNA extraction, stimulated BMDMs were resuspended in TRIzol Reagent and stored at –80°C until use.

### qRT-PCR.

Total RNAs were quantified using a NanoDrop ND-1000 spectrophotometer. RNA was reverse transcribed using Evo M-MLV RT Master Mix (Thermo Fisher Scientific, 28025013). qRT-PCR was performed on an ABI 7500 PCR system (Thermo Fisher Scientific) using the SYBR Green Master Mix (Bio-Rad, 1725121). Gene expression was calculated in 2^–ΔΔCt^. Expression of the target genes was normalized to GAPDH and displayed as fold change relative to control groups. The specific primers used in PCR are listed in Supplemental Table 3.

### RNA-Seq.

Mouse mammary tumor total RNA was isolated by using the Quick-DNA/RNA Microprep Plus kit per the product’s manual (Zymo Research, D7005); samples were sent to Novogene Co for RNA-Seq. Each group contained 4 biological replicates. RNA samples were quantified and qualified using the Agilent 2100 Bioanalyzer (Agilent Technologies) and ABI StepOnePlus Real-Time PCR System (Thermo Fisher Scientific) and sequenced using Illumina HiSeq 2000. RNA-Seq data were analyzed using Pipeline v5.0. Raw sequencing data were demultiplexed and examined for high quality. Then, differentially expressed genes were screened using the limma package (threshold: |log_2_ fold change| ≥ 1, adjusted *P* < 0.05) and further analyzed by clusterProfiler, DOSE, GO.db and topGO packages. Original data are available at the National Center for Biotechnology Information (NCBI) Gene Expression Omnibus (GEO) (accession GSE220667).

### MTS-based cell proliferation assay.

WT and *IL20rb*^–/–^ PyMt cells were plated at a density range of 1 × 10^5^ to 1 × 10^6^ cells per well in 96-well plates with 200 μL DMEM and incubated for 72 hours in a 37°C incubator with 5% CO_2_ atmosphere. Cell proliferation analysis was performed by using a CellTiter 96 AQueous One Solution Cell Proliferation Assay kit (Promega, G3580). 20 μL of the CellTiter 96 AQueous One Solution Reagent was added to each well, followed by incubation at 37°C for 1 hour in a 5% CO_2_ humidified atmosphere. Optical density at 562 nm was subsequently measured using the Synergy Neo2 (BioTek).

### ELISA.

Serum TSLP and HMGB1 levels were determined using a Max Mouse TSLP ELISA kit (BioLegend, 434107) and mouse HMGB1 ELISA kit (Thermo Scientific, EEL102) according to the manufacturer’s instructions. Optical densities were measured on Synergy Neo2 (BioTek) at 450 nm, and cytokine concentrations were calculated by a 5-parameter logistic curve using Gen5 Microplate Reader and Imager Software (BioTek).

### TCGA cohorts’ survival analyses.

The phenotype dataset (with survival outcomes) and the RNA-Seq data of the breast cancer were downloaded through R package “UCSCXenaTools” (51). Then, the count data were transformed into the transcripts per kilobase million values by the “count2tpm” function implemented in the R package “IOBR” (52). The Kaplan-Meier curves were used for overall survival, progression-free interval, disease-specific survival, and disease-free interval. The log-rank test applied to test the survival difference between high and low *IL24* expression was performed by R package “survival.”

### Statistics.

Graphs and statistical analysis were generated using Prism 9.0 (GraphPad Software Inc.). Statistical significance for values between independent groups was determined by Mann-Whitney *U* test. One-way ANOVA with Tukey’s multiple comparisons test was used for multiple group comparisons. Two-way ANOVA with repeated measures was used to compare the tumor growth curves. Correlations between parameters were assessed by Spearman’s rank correlation analysis. Two-tailed Fisher’s exact test was used to compare HMGB1 distribution in tumor versus stromal cells. Log-rank test was used for survival plots. The number of individual data points shown in each graph represents independent biological replicates. Two-tailed *P* values of less than 0.05 were considered significant.

### Study approval.

All animal studies were reviewed and approved by the Massachusetts General Hospital Institutional Animal Care and Use Committee.

### Data availability.

The RNA-Seq data can be accessed from the NCBI database (GEO accession GSE220667). Values for all data points found in graphs can be found in the supplemental supporting data values file. Additional data related to this paper may be requested from the corresponding author.

## Author contributions

SD conceived the study. BW, YX, and SD designed the experiments. BW, YX, CZ, YZ, and HGS performed the experiments and analyzed the data. BW, YX, CZ, YZ, and SD interpreted the data. BW, YX, and SD wrote the manuscript.

## Supplementary Material

Supplemental data

Supporting data values

## Version 1 01/09/2025

Electronic publication

## References

[1] Pilipow K (2021). T-cell-based breast cancer immunotherapy. Semin Cancer Biol.

[2] Sorlie T (2001). Gene expression patterns of breast carcinomas distinguish tumor subclasses with clinical implications. Proc Natl Acad Sci U S A.

[3] Hamilton PT (2022). Tumour immunotherapy: lessons from predator-prey theory. Nat Rev Immunol.

[4] Valsecchi ME (2015). Combined nivolumab and ipilimumab or monotherapy in untreated melanoma. N Engl J Med.

[5] Vonderheide RH (2017). Immunotherapy for breast cancer: what are we missing?. Clin Cancer Res.

[6] Pardoll DM (2012). The blockade of immune checkpoints in cancer immunotherapy. Nat Rev Cancer.

[7] Shan F (2022). Therapeutic targeting of regulatory T cells in cancer. Trends Cancer.

[8] Oh DY, Fong L (2021). Cytotoxic CD4^+^ T cells in cancer: expanding the immune effector toolbox. Immunity.

[9] Chraa D (2019). T lymphocyte subsets in cancer immunity: friends or foes. J Leukoc Biol.

[10] Aspord C (2013). Plasmacytoid dendritic cells support melanoma progression by promoting Th2 and regulatory immunity through OX40L and ICOSL. Cancer Immunol Res.

[11] Demehri S (2016). Thymic stromal lymphopoietin blocks early stages of breast carcinogenesis. J Clin Invest.

[12] Hung K (1998). The central role of CD4(+) T cells in the antitumor immune response. J Exp Med.

[13] Boieri M (2022). CD4+ T helper 2 cells suppress breast cancer by inducing terminal differentiation. J Exp Med.

[14] Binnewies M (2019). Unleashing type-2 dendritic cells to drive protective antitumor CD4^+^ T cell immunity. Cell.

[15] Oh DY (2020). Intratumoral CD4^+^ T cells mediate anti-tumor cytotoxicity in human bladder cancer. Cell.

[16] Mantovani A (2022). Macrophages as tools and targets in cancer therapy. Nat Rev Drug Discov.

[17] Noy R, Pollard JW (2014). Tumor-associated macrophages: from mechanisms to therapy. Immunity.

[18] Murray PJ (2014). Macrophage activation and polarization: nomenclature and experimental guidelines. Immunity.

[19] Mantovani A, Sica A (2010). Macrophages, innate immunity and cancer: balance, tolerance, and diversity. Curr Opin Immunol.

[20] Tzetzo SL, Abrams SI (2021). Redirecting macrophage function to sustain their “defender” antitumor activity. Cancer Cell.

[21] Fontana F (2021). N-cadherin in osteolineage cells modulates stromal support of tumor growth. J Bone Oncol.

[22] Meyer MA (2018). Breast and pancreatic cancer interrupt IRF8-dependent dendritic cell development to overcome immune surveillance. Nat Commun.

[23] Liu C (2021). Advances in rodent models for breast cancer formation, progression, and therapeutic testing. Front Oncol.

[24] Menezes ME (2014). MDA-7/IL-24: multifunctional cancer killing cytokine. Adv Exp Med Biol.

[25] Tang D (2023). The multifunctional protein HMGB1: 50 years of discovery. Nat Rev Immunol.

[26] Xie S (2023). Novel tripeptide RKH derived from *Akkermansia muciniphila* protects against lethal sepsis. Gut.

[27] Sauane M (2006). N-glycosylation of MDA-7/IL-24 is dispensable for tumor cell-specific apoptosis and “bystander” antitumor activity. Cancer Res.

[28] Ribas A, Wolchok JD (2018). Cancer immunotherapy using checkpoint blockade. Science.

[29] Cunningham TJ (2017). Randomized trial of calcipotriol combined with 5-fluorouracil for skin cancer precursor immunotherapy. J Clin Invest.

[30] Schmitt CA (2011). Immunotherapy: TSLP fuels inflammation in breast and pancreatic tumors. Nat Rev Clin Oncol.

[31] Kuan EL, Ziegler SF (2018). A tumor-myeloid cell axis, mediated via the cytokines IL-1α and TSLP, promotes the progression of breast cancer. Nat Immunol.

[32] Bluestone JA (2009). The functional plasticity of T cell subsets. Nat Rev Immunol.

[33] Malandro N (2016). Clonal abundance of tumor-specific CD4(+) T cells potentiates efficacy and alters susceptibility to exhaustion. Immunity.

[34] Johansson M (2008). Polarized immune responses differentially regulate cancer development. Immunol Rev.

[35] Kim H (2022). Calcipotriol, a synthetic vitamin D analog, promotes antitumor immunity via CD4+T-dependent CTL/NK cell activation. Biomed Pharmacother.

[36] Whitaker EL (2012). Interleukin 24: mechanisms and therapeutic potential of an anti-cancer gene. Cytokine Growth Factor Rev.

[37] Pestka S (2004). Interleukin-10 and related cytokines and receptors. Annu Rev Immunol.

[38] Sauane M (2003). MDA-7/IL-24: novel cancer growth suppressing and apoptosis inducing cytokine. Cytokine Growth Factor Rev.

[39] Jiang H (1995). Subtraction hybridization identifies a novel melanoma differentiation associated gene, mda-7, modulated during human melanoma differentiation, growth and progression. Oncogene.

[40] Dabitao D (2018). Cell-specific requirements for STAT proteins and type I IFN receptor signaling discretely regulate IL-24 and IL-10 expression in NK cells and macrophages. J Immunol.

[41] Huang EY (2001). Genomic structure, chromosomal localization and expression profile of a novel melanoma differentiation associated (mda-7) gene with cancer specific growth suppressing and apoptosis inducing properties. Oncogene.

[42] Su ZZ (1998). The cancer growth suppressor gene mda-7 selectively induces apoptosis in human breast cancer cells and inhibits tumor growth in nude mice. Proc Natl Acad Sci U S A.

[43] Jiang H (1996). The melanoma differentiation associated gene mda-7 suppresses cancer cell growth. Proc Natl Acad Sci U S A.

[44] Menezes ME (2015). MDA-7/IL-24 functions as a tumor suppressor gene in vivo in transgenic mouse models of breast cancer. Oncotarget.

[45] Li YJ (2015). Suppression of Her2/Neu mammary tumor development in mda-7/IL-24 transgenic mice. Oncotarget.

[46] Sarkar D (2005). Dual cancer-specific targeting strategy cures primary and distant breast carcinomas in nude mice. Proc Natl Acad Sci U S A.

[47] Dash R (2014). Novel mechanism of MDA-7/IL-24 cancer-specific apoptosis through SARI induction. Cancer Res.

[48] Jiao Y (2007). Growth suppression and radiosensitivity increase by HMGB1 in breast cancer. Acta Pharmacol Sin.

[49] Yang M (2022). Negative effects of stromal neutrophils on T cells reduce survival in resectable urothelial carcinoma of the bladder. Front Immunol.

[50] Roehle K (2021). cIAP1/2 antagonism eliminates MHC class I-negative tumors through T cell-dependent reprogramming of mononuclear phagocytes. Sci Transl Med.

[51] Wang S, Liu X (2019). G3F: global, multidimensional spectral regression analysis. J Open Source Softw.

[52] Zeng D (2021). IOBR: multi-omics immuno-oncology biological research to decode tumor microenvironment and signatures. Front Immunol.

