# Supplementary material for: CD4+ T helper 2 cell–macrophage crosstalk induces IL-24–mediated breast cancer suppression
Source: JCI Insight. 2025 Jan 9;10(1):e180962. doi: 10.1172/jci.insight.180962 (PMC11721301; doi:10.1172/jci.insight.180962)
Supplement: Supplemental data [file jciinsight-10-180962-s270.pdf]

Supplemental Data

CD4$^+$ T helper 2 cell-macrophage crosstalk induces interleukin 24-mediated breast cancer suppression

Bo Wang, Yun Xia, Can Zhou, Yuhan Zeng, Heehwa G. Son, Shadmehr Demehri*

*Author for correspondence:

Shadmehr Demehri, M.D., Ph.D.

Email: sdemehri1@mgh.harvard.edu

Supplemental Figures

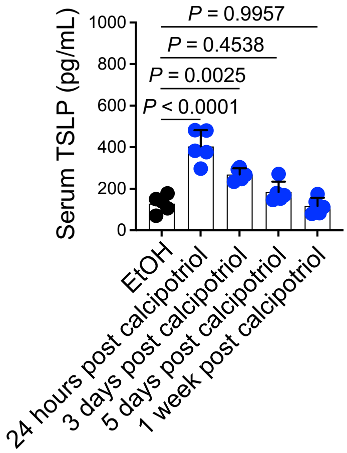

Supplemental Figure 1. Topical calcipotriol treatment increases circulating TSLP levels. Six-week-old wild-type (WT) mice were injected with PyMt cells into the mammary fat pad. Starting two days after orthotopic PyMt tumor cell implantation, the mice were treated topically with 20 nmol calcipotriol every two days. TSLP levels in circulation were measured following the cessation of calcipotriol treatment (n=5 in each group). EtOH solvent was used in the control group. Bar graph shows mean + SD, one-way ANOVA.

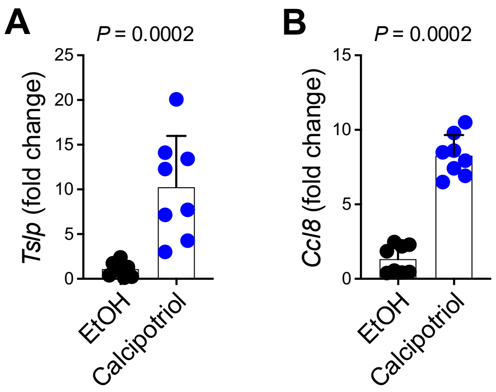

Supplemental Figure 2. qRT-PCR validation of *Tslp* and *Ccl8* induction in calcipotriol-treated tumors. (**A** and **B**) Gene expression analysis of (**A**) *Tslp* and (**B**) *Ccl8* in calcipotriol-treated versus EtOH-treated tumors (n = 8 in each group). Results are expressed as fold change compared with EtOH control. Bar graphs show mean + SD, Mann-Whitney *U* test (A and B).

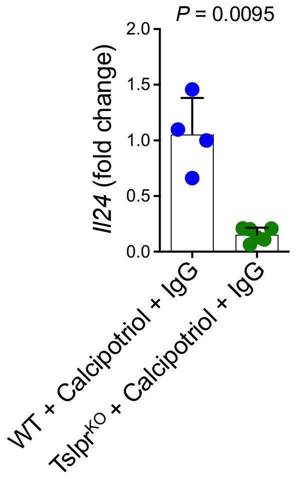

Supplemental Figure 3. *Il24* induction by calcipotriol in mammary tumors implanted in WT and Tslpr^{KO} mice. *Il24* expression in PyMt tumors grown in WT versus Tslpr^{KO} mice treated with calcipotriol. Groups: WT + Calcipotriol + IgG (n = 4), Tslpr^{KO} + Calcipotriol + IgG (n = 6). Bar graph shows mean + SD, Mann-Whitney *U* test.

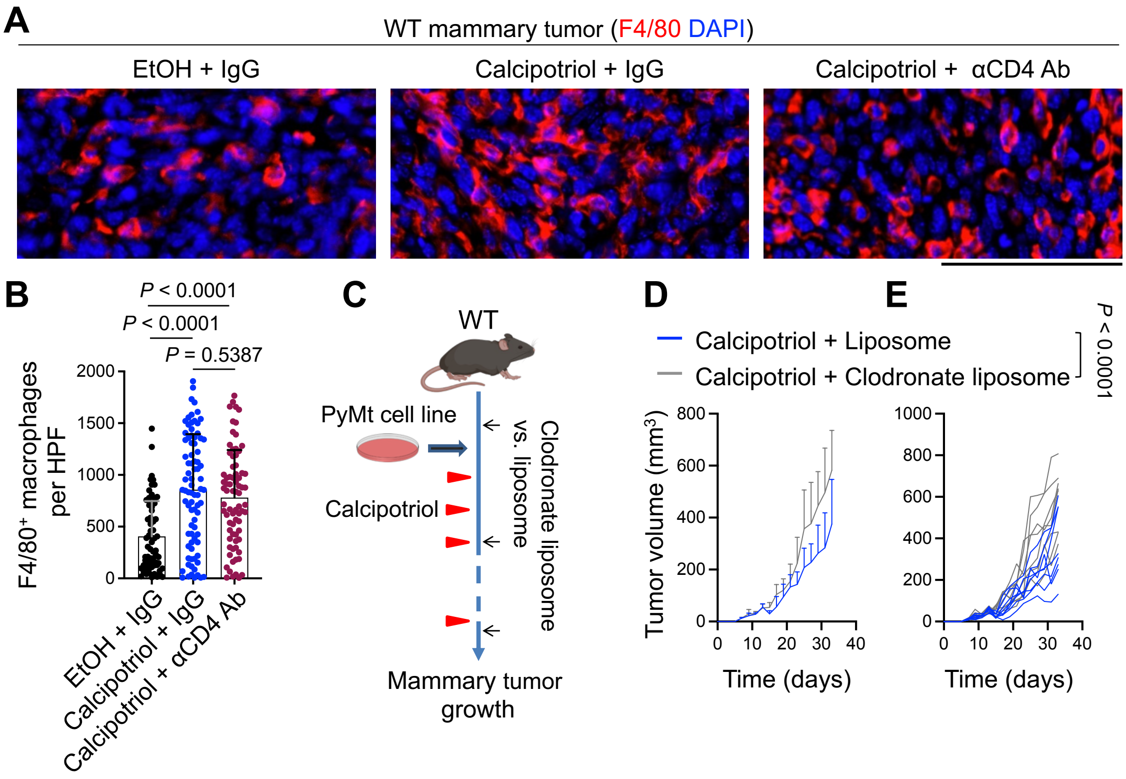

Supplemental Figure 4. Macrophages are essential for the therapeutic effect of calcipotriol on mammary tumors. (**A**) Representative images of F4/80 stained PyMt mammary tumor in WT mice treated with EtOH + IgG, calcipotriol + IgG, or calcipotriol + αCD4 Ab. (**B**) Quantification of F4/80$^+$ macrophages in WT mammary tumors treated with EtOH + IgG (n = 6), calcipotriol + IgG (n = 7), or calcipotriol + αCD4 Ab (n = 7). F4/80$^+$ macrophages were counted in up to ten randomly selected HPF images from each tumor. Each dot indicates F4/80$^+$ macrophage count in one HPF image. (**C**) Schematic diagram of the experimental setup to assess the contribution of macrophages to the efficacy of topical calcipotriol treatment against PyMt mammary tumor growth. (**D** and **E**) PyMt mammary tumor volume in WT animals treated by topical calcipotriol with liposome (control) versus clodronate liposome shown as (D) mean tumor volumes + SD and (E) spider plot of mammary tumor volume over time (n = 8 in each group). Bar graph shows mean + SD, one-way ANOVA (B), two-way ANOVA (D and E), scale bar: 100 µm (A).

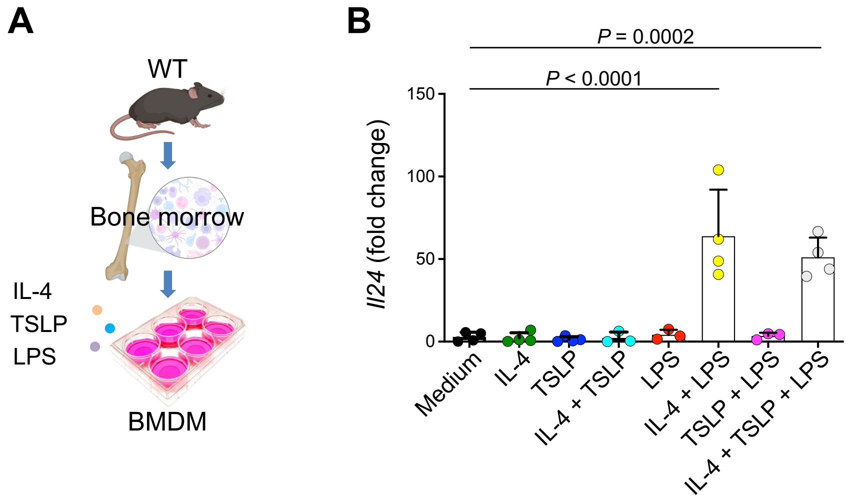

Supplemental Figure 5. IL-4 plus TLR4 agonist induces IL-24 expression in BMDMs. (**A**) Schematic diagram of the experimental design to evaluate *Il24* induction in WT BMDMs after stimulation with IL-4, TSLP, LPS, or their combinations. (**B**) *Il24* mRNA levels 3 hours after exposure to the indicated stimulations. Groups: Medium (n = 4), IL-4 (n = 4), TSLP (n = 4), IL-4 + TSLP (n = 3), LPS (n = 3), IL-4 + LPS (n = 4), TSLP + LPS (n = 3), and IL-4 + TSLP + LPS (n = 4). Bar graph shows mean + SD, one-way ANOVA.

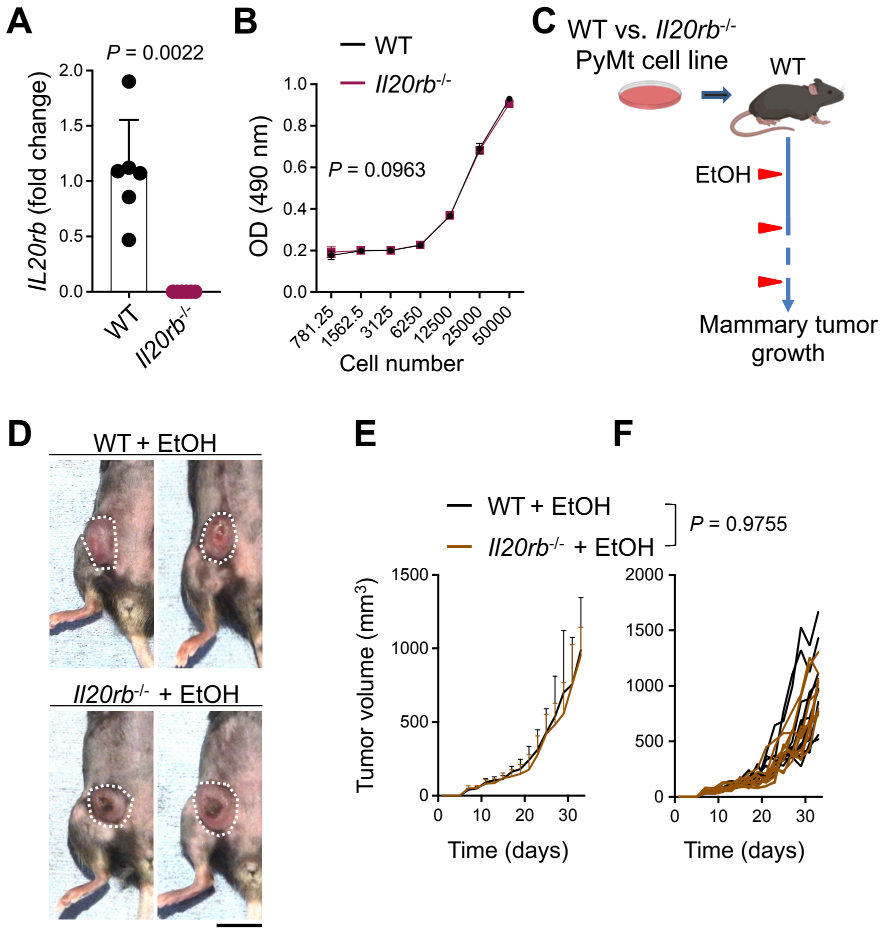

Supplemental Figure 6. Growth rates of WT and *Il20rb*$^{-/-}$ PyMt mammary tumor cells *in vitro* and *in vivo*. (**A**) *IL20rb* expression in WT versus *IL20rb*$^{-/-}$ PyMt cells (n = 6 in each group). (**B**) The proliferation rate of WT versus *IL20rb*$^{-/-}$ PyMt cells *in vitro* using an MTS assay (n = 5 in each group). (**C**) Schematic diagram outlining the experimental design used in EtOH control study to test the role of IL-24/IL-20R axis in mediating the antitumor impact of topical calcipotriol treatment on PyMt mammary tumor growth. Six- to eight-week-old WT mice were used as tumor recipients. The animals were treated with 20 nmol calcipotriol versus EtOH topically every 2 days, starting at 2 days after orthotopic WT or *Il20rb*$^{-/-}$ PyMt mammary tumor cell implantation. Calcipotriol treatment groups are presented in Figure 5. (**D**) Representative macroscopic images of EtOH-treated mammary tumors at the endpoint (dotted circles highlight the tumor sites). (**E** and **F**) WT and *Il20rb*$^{-/-}$ PyMt mammary tumor volume in WT animals treated with topical EtOH shown as (E)

mean tumor volumes + SD and (F) spider plot of mammary tumor volume over time (n = 10 in each group). Bar graphs show mean + SD, Mann-Whitney *U* test (A), and two-way ANOVA (B, E, and F), scale bar: 1cm (D).

Supplemental Table 1. Univariate and multivariate analyses of factors associated with overall survival in TCGA-BRCA patients (*n* = 853).

	Univariate			Multivariate		
**Variable**	**HR**	**95% CI**	***P*-value**	**HR**	**95% CI**	***P*-value**
Age, years	1.028	1.014-1.043	**<0.001**	1.030	1.014-1.046	**<0.001**
IL24 (high vs. low)	0.527	0.360-0.771	**0.001**	0.651	0.429- 0.986	**0.043**
T Stage (T_{3+4} vs. T_{1+2})	1.716	1.130-2.605	**0.011**	1.248	0.768- 2.029	0.372
N Stage (N+ vs. N-)	2.588	1.684-3.977	**<0.001**	2.407	1.503- 3.854	**<0.001**
M Stage (III-IV vs. I-II)	7.546	4.285-13.289	**<0.001**	3.496	1.801- 6.787	**<0.001**
Gender (Male vs. Female)	0.795	0.111-5.700	0.8191			**NA**

Abbreviations: TCGA, The Cancer Genome Atlas; BRCA, breast cancer; HR, hazard ratio; CI, confidence interval; NA, not applicable. T Stage, Tumor stage; N stage, Nodal Stage; M stage, metastasis stage.

Variables associated with overall survival in the univariate analysis were adopted as covariates the multivariate Cox regression analysis. Significant *P*-values are shown in **bold**. HR > 1 = increased risk; HR < 1 = decreased risk.

Supplemental Table 2. Antibodies used in the study.

**Immunofluorescence Antibodies**	**Conjugate**	**Clone**	**Manufacturer**	**Cat #**	**Isotype**	**RRID**
CD3	Purified	CD3-12	Abcam	Ab11089	Rat IgG1	AB_369097
CD4	Purified	EPR19514	Abcam	Ab183685	Rabbit IgG	AB_2686917
CD8α	Purified	D4W2Z	Cell Signaling Technologies	98941	Rabbit IgG	AB_2756376
HMGB1	Purified	D3E5	Cell Signaling Technologies	6893	Rabbit IgG	AB_10827882
F4/80	Purified	D2S9Rl	Cell Signaling Technologies	70076S	Rabbit IgG	AB_2799771
IL-24	Purified	303308	R&D Systems	MAB2786-SP	Rat IgG2A	AB_10890924
Ly6G	Purified	E6Z1T	Cell Signaling Technologies	87048	Rabbit IgG	AB_2909808
Cleaved Caspase-3	Purified	5A1E	Cell Signaling Technologies	9664	Rabbit IgG	AB_2070042
Cytokeratin	Purified	AE1/AE3	Dako	M3515	Mouse IgG1 kappa	AB_2132885
**Blocking Antibodies**	**Conjugate**	**Clone**	**Manufacturer**	**Cat #**	**Isotype**	**RRID**
*InVivo*MAb anti-mouse CD4	Purified	GK1.5	BioXCell	BE0003	Rat IgG2b, κ	AB_1107636
Rat IgG2b-UNLB	Purified	SB64a	SouthernBiotech	0127-01	Rat IgG2b	AB_2794066
**Secondary Antibodies**	**Conjugate**	**Clone**	**Manufacturer**	**Cat #**	**Isotype**	**RRID**
Goat anti-Rabbit IgG	Alexa Fluor™ 568	Polyclonal	Thermofisher Scientific	A11036	Goat IgG	AB_10563566
Goat Anti-Rat IgG	Alexa Fluor™ 647	Polyclonal	Thermofisher Scientific	A21247	Goat IgG	AB_141778
Goat Anti-Rat IgG	Alexa Fluor™ 488	Polyclonal	Thermofisher Scientific	A11006	Goat IgG	AB_2534074
Goat Anti-Mouse IgG	Alexa Fluor™ 568	Polyclonal	Thermofisher Scientific	A11004	Goat IgG	AB_2534072

Supplemental Table 3. PCR primer sets used in the study.

**Name**	**Sequence**(*5′ −3′)*
Il24 F	CAATTCCATGCTTCCCATTAGTG
Il24 R	*ATTTCTGCATCCAGGTCAGGAG*
Il20rb F	*ACCCCTTTAACCGAAATGCAA*
Il20rb R	*TTAACATGTTCCGCGGCGCC*
Tslp F	CCAGGCTACCCTGAAACTGA
Tslp R	TCTGGAGATTGCATGAAGGA
Ccl8 F	TCTACGCAGTGCTTCTTTGCC
Ccl8 R	AAGGGGGATCTTCAGCTTTAGTA
